# Cerebrovascular function in tension-type headache and migraine with or without aura: Transcranial Doppler study

**DOI:** 10.1038/s41598-022-18359-6

**Published:** 2022-08-18

**Authors:** Eman M. Khedr, Mohammed A. Abbas, Ayman Gamea, Mohamed A. Sadek, Ahmed F. Zaki

**Affiliations:** 1 Department of Neuropsychiatry, Faculty of Medicine, Assiut University Hospital, Assiut, Egypt; 2 Neuropsychiatric Department, Faculty of Medicine, Aswan University Hospital, Aswan, Egypt; 3 Neuropsychiatry Department, Faculty of Medicine, Luxor University, Luxor, Egypt; 4 Neuropsychiatric Department, Faculty of Medicine, South Valley University, Qena University Hospital, Qena, Egypt

**Keywords:** Diseases, Neurology

## Abstract

The aim of the current study was to determine whether tension-type headache (TTH) and migraine with or without aura have altered anterior and posterior circulation compared with normal volunteers as assessed by Transcranial Doppler (TCD) ultrasonography. The study included 24 patients with chronic TTH and 37 patients with migraine (16 with aura and 21 without aura) classified according to the diagnostic criteria of the International Headache Society 2018. They were compared with a control group of 50 age- and sex-matched healthy volunteers. Each participant was examined with TCD ultrasonography of the middle, anterior and posterior cerebral and vertebral arteries (MCA, ACA, PCA, and VA) at rest. Patients in the TTH group had a significantly lower peak systolic velocity (PSV) and mean flow velocity (MFV) in the MCA compared with controls, whereas EDV and MFV in the ACA were significantly higher in the migraine without aura group than controls. Within the 3 groups of patients, the TTH group had significantly lower PSV in the MCA and PCA than the group of migraine with aura. In addition, the TTH group had significantly lower PSV and MFV in the MCA and a lower EDV in the VA than migraine patients without aura. In conclusion, the possibility of cerebrovascular changes is confirmed in the present study in both TTH and migraine without aura. The former has a low MFV in the MCA whereas the latter has a high MFV in the ACA.

## Introduction

Transcranial Doppler (TCD) is a noninvasive technique that uses a pulsed ultrasonic beam to evaluate the velocity, direction, and other properties of blood flow in the cerebral arteries, as well as the cerebrovascular reserve. The flow velocities measured with TCD are directly proportional to invasive flow measurements^1,2^. Migraine is a disabling neurovascular disorder that affects around 12% of the general population, and in approximately one third of these patients, migraine attacks are preceded by neurological symptoms associated with a transient cortical malfunction, collectively known as aura^3^. The mechanisms underlying migraine pain remain elusive, and both vascular and neural mechanisms have been investigated and discussed. However, the vascular hypothesis of vasodilatation has dominated migraine research for most of the twentieth century^4^.

Ozkalayci et al., found no significant differences in flow velocity of migraine patients with or without aura during a headache-free episode when compared with controls or to each other^2^. Frieberg reported reduced CBF velocities in the MCA of the headache side during a migraine attack when compared to data from the non-headache side that had been acquired during an attack-free episode^5^. In contrast, Zwetsloot reported no velocity changes in the MCA of either the headache and non-headache side MCAs during an acute attack^6^. A recent meta-analysis found that migraineurs have a higher resting mean blood flow velocity (MFV) in both anterior and posterior circulations than controls and it was argued that this could be a hallmark of migraine^7^. An increase in CBF could be due to a decrease in the cross-sectional area of a vessel at or near the point of insonation or to regional flow changes at the level of arterioles^8^.

Several studies have used TCD sonography to study migraine patients^8–20^. However few studies have been performed on patients with TTH or have directly compared TTH and migraine patients^21–25^ and few have emphasized the importance of vascular factors in TTH^26–29^. For example, Wallasch reported increased CBF velocities in the MCA, ACA and PCA as well as decreased pulsatile index in patients with episodic tension-type headache, while no difference was observed in patients with chronic TTH^25^. But there have been no subsequent comparable studies. In fact, there is evidence from previous studies that TTH and migraine headache may not be discrete pathophysiologic entities but form a continuum^30^ which may make it difficult to distinguish changes between groups.

Due to the contradictory results of interictal hemodynamic abnormalities detected by TCD ultrasonography in migraine and the rarity of studies in TTH the aim of the present study is to re-evaluate cerebral hemodynamics in TTH and migraine patients in a large sample and compare the results with normal volunteers.

## Results

Table 1 showed the demographic and clinical data of each group. There were no significant differences between groups in mean age or sex distribution. There was no significant difference between patient groups in duration of illness. The frequency of attacks was significantly higher in TTH group and family history was significantly more common among migraine with aura than other groups.

**Table 1:** Demographic and clinical data of studied groups.

Variables/group	Control	Tension-type headache	Migraine with aura	Migraine without aura	P value between different groups
Number of patients	50	24	16	21	
Age (mean ± SD)	31.4 ± 8.3	35.5 ± 7.4	30.12 ± 7.4	31 ± 9.1	0.119
Sex Male/female	27/23	8/16	9/7	9/12	0.097
Duration of disease in months (mean ± SD)	–	33.7 ± 33.8	32.25 ± 21.7	33.9 ± 23.63	0.981
Frequency of attacks/month (mean ± SD)	–	20.33 ± 4.5	11.06 ± 4.7^^^	12.9 ± 4.1^^^	0.001
Family history	–	5 (20.8%)	13 (81.25%)^^^	11 (52.4%)^^^	0.001

P value is significant < 0.05, between groups, ^^^Migraine group versus tension headache group.

Table 2 showed the comparison between subgroups of headache according to the frequency of attacks (episodic tension-type headache versus chronic tension-type headache and episodic migraine versus chronic migraine) compares the duplex parameters of anterior (MCA and ACA) and posterior circulation (PCA and VA). There were no significant differences between subgroups of the same type of headache except in pulsatility index which was significantly higher in chronic migraine compared with episodic migraine in both MCA and PCA.

**Table 2:** Differences in the Resting duplex parameters of anterior and posterior cerebral circulations between different subgroups of each type of headache (chronic tension type versus episodic tension type headache) and subgroups of migraine (chronic migraine versus episodic migraine).

Artery	Parameter	Chronic tension type headache Mean ± SD (16 cases)	Episodic tension type headache Mean ± SD (8 cases)	Chronic tension-type headache versus episodic tension-type headache	Episodic migraine (23 cases)	Chronic migraine (14 cases)	Episodic migraine vs chronic migraine
		Mean ± SD		P value, F, t, df	Mean ± SD		P value, F, t, df
MCA	PSV	64.92 ± 14.32	76.84 ± 62	0.115, 2.91, − 1.64, 22	83.47 ± 20.67	83.65 ± 28.00	0.98, 2.48, − 0.02, 35
	EDV	29.72 ± 7.63	36.08 ± 14.25	0.16, 3.53, − 1.43, 22	38.70 ± 11.36	35.92 ± 12.82	0.49, 1.13, 0.68, 35
	Mean	41.46 ± 9.39	49.61 ± 16.31	0.129, 3.87, − 1.57, 22	53.62 ± 14.20	51.83 ± 17.47	0.73, 2.11, 0.34, 35
	PI	1.04 ± 1.06	0.74 ± 0.09	0.439, 1.54, 0.78, 22	0.8217 ± 0.17	0.9321 ± 0.10	0.02*, 4.09, − 2.16, 35
ACA	PSV	56.39 ± 11.57	67.52 ± 15.04	0.050, 1.40, − 2.19, 22	62.57 ± 14.08	59.92 ± 14.72	0.58, 0.21, 0.54, 35
	EDV	25.35 ± 8.41	30.96 ± 7.93	0.131,0.01, − 1.56, 22	26.24 ± 5.49	25.15 ± 7.31	0.60, 1.68, 0.51, 35
	Mean	35.70 ± 8.38	43.50 ± 10.00	0.056, 0.79, − 2.01, 22	38.34 ± 7.33	36.74 ± 9.16	0.56, 0.605, 0.58, 35
	PI	0.89 ± 0.24	0.93 ± 0.17	0.67, 0.91, − 0.42, 22	0.85 ± 0.22	0.91 ± 0.14	0.41, 1.557, − 0.83, 35
PCA	PSV	49.31 ± 13.98	52.60 ± 10.81	0.56, 0.96, − 0.58, 22	57.12 ± 11.23	57.50 ± 8.71	0.913, 0.210, − 0.11, 35
	EDV	22.46 ± 7.4	24.87 ± 6.79	0.45, 0.44, − 0.76, 22	27.79 ± 7.35	24.81 ± 6.15	0.213, 0.350, 1.26, 35
	Mean	31.41 ± 9.40	34.12 ± 8.05	0.49, 0.65, − 0.69, 22	37.81 ± 8.48	35.71 ± 6.65	0.435, 0.429, 0.790, 35
	PI	0.86 ± 0.25	0.71 ± 0.06	0.12, 9.78, 1.61, 22	0.75 ± 0.13	0.89 ± 0.11	0.002*, 0.762, − 3.27, 35
VA	PSV	45.93 ± 11.95	46.54 ± 15.30	0.91, 1.46, − 0.10, 22	49.98 ± 14.03	50.89 ± 12.00	0.841, 0.747, − 0.20, 35
	EDV	19.53 ± 4.97	19.19 ± 5.08	0.87, 0.21, 0.156, 22	22.47 ± 6.76	21.73 ± 4.08	0.714, 1.679, 0.36, 35
	Mean	28.33 ± 6.09	28.31 ± 8.18	0.99, 1.31, 0.007, 22	32.33 ± 8.52	31.45 ± 5.98	0.738, 1.491, 0.33, 35
	PI	0.83 ± 0.21	0.91 ± 0.29	0.436, 2.51, − 0.79, 22	0.8839 ± 0.21	0.90 ± 0.14718	0.806, 1.052, − 0.247, 35

*MCA* middle cerebral artery, *ACA* anterior cerebral artery, *PCA* posterior cerebral artery, *VB* vertebral artery, *PSV* Peak Systolic velocity, *EDV* End Diastolic Velocity, *PI* pulsatility Index, *mean* mean flow velocity, P value is significant < 0.05, *the significant between each subgroup of the same type of headache.

Table 3 showed the comparison between controls and each type of headache in different parameters of ultrasonography of the 4 vessels. There were significant differences between the 4 groups in PSV and MFV of the MCA, and EDV of ACA, with Bonferroni correction these significant were due to significant lower PSV and MFV of MCA of the TTH group than controls whereas EDV, and MFV of the ACA were significantly higher in migraine without aura than controls.

**Table 3:** Differences in the Resting duplex parameters of anterior and posterior cerebral circulations between control group versus each type of headache (tension headache and migraine with and without aura).

Artery	Parameters	Controls	Tension-type headache	Migraine with aura		Migraine without aura	P value between four groups (ANOVA)	P value controls versus tension-type headache	P value controls versus migraine with aura		P value controls versus migraine without aura
		Mean ± SD					P value, F, df	P value, F, t, df			
MCA	PSV	88.1 ± 2.17	68.9 ± 1.73	85.7 ± 2.8		81.9 ± 1.96	0.006*, 4.44, 3	< 0.0001*, 2.41, − 3.7, 72	0.75, 1.53, − 0.35, 64		0.24, 0.78, − 1.12, 69
	EDV	35.8 ± 1.14	31.8 ± 10.5	38.9 ± 1.45		36.6 ± 9.4	0.24, 1.39, 3	0.14, 0.90, − 1.43, 72	0.43, 2.61, 0.90, 64		0.74, 0.69, 0.29, 69
	Mean	53.1 ± 14.3	44.2 ± 12.4	55.0 ± 18.4		50.2 ± 16.1	0.05*, 2.65, 3	0.006*, 0.78, − 2.74, 72	0.80, 3.10, 0.28, 64		0.64, 0.18, − 0.450, 69
	PI	0.89 ± 0.10	0.78 ± 0.12	0.89 ± 0.10		0.84 ± 0.25	0.90, 0.19, 3	0.80, 5.47, 0.36, 72	0.22, 2.26, − 1.55, 64		0.69, 2.37, − 0.46, 69
ACA	PSV	59.3 ± 1.35	60.4 ± 13.8	58.3 ± 1.57		64.01 ± 1.26	0.56, 0.68, 3	0.75, 0.08, 0.312, 72	0.80, 0.001, − 0.25, 64		0.17, 0.98 , 1.33, 69
	EDV	23.4 ± 6.4	27.0 ± 8.7	23.7 ± 4.7		27.42 ± 6.74	0.04*, 2.83, 3	0.06, 3.29, 2.14, 72	0.83,1.46, 0.18, 64		0.02*, 0.09, 2.364, 69
	Mean	35.4 ± 8	38.5 ± 9.7	35.2 ± 7.8		39.6 ± 7.7	0.179, 1.66, 3	0.21, 0.42, 1.32, 72	0.92, 0.168, − 0.09, 64		0.04*, 0.89, 1.99, 69
	PI	0.91 ± 0.14	0.90 ± 0.22	0.83 ± 0.18		0.91 ± 0.21	0.46, 0,0.86, 3	0.91, 5, − 0.11, 72	0.12, 0.20, − 1.77, 64		0.96, 1.77, 0.05, 69
PCA	PSV	55.8 ± 1.4	50.4 ± 1.29	58.9 ± 1.21		55.98 ± 8.54	0.178, 1.669, 3	0.10, 0.99, − 1.59, 72	0.40, 0.70, 0.78, 64		0.96, 5.52, 0.03, 69
	EDV	24.4 ± 7.9	23.3 ± 7.2	27.1 ± 7.7		26.29 ± 6.5	0.332, 1.152, 3	0.51, 0.25, − 0.62, 72	0.23, 0.001, 1.18, 64		0.32, 0.25, 0.92, 69
	Mean	34.5 ± 9.5	32.3 ± 8.9	38 ± 9.19		36.1 ± 6.7	0.201, 1.570, 3	0.36, 0.53, − 0.89, 72	0.17, 0.01, 1.36, 64		0.37, 3.12, 0.78, 69
	PI	0.84 ± 0.13	0.81 ± 0.22	0.811 ± 0.12		0.81 ± 0.16	0.761, 0.389, 3	0.52, 7.54, − 0.76, 72	0.39, 0.004, − 0.84, 64		0.38, 1.62, − 0.95, 69
VA	PSV	49.7 ± 1.09	46.1 ± 12.8	48.3 ± 9.96		51.8 ± 1.51	0.441, 0.905, 3	0.24, 0.48, − 1.25, 72	0.63, 0.330, − 0.46, 64		0.56, 3.61, 0.66, 69
	EDV	20.2 ± 6.2	19.4 ± 4.9	20.8 ± 3.6		23.2 ± 6.9	0.147, 1.824, 3	0.52,0.88, − 0.58, 72	0.67, 3.16, 0.323, 64		0.09, 0.73, 1.77, 69
	Mean	30.4 ± 7.2	28.3 ± 6.7	30.1 ± 4.8		32.7 ± 9.1	0.228, 1.468, 3	0.32, 0.8, − 0.97, 72	0.56, 1.910, 0.471, 64		0.23, 1.47, 1.336, 69
	PI	1.3 ± 1.9	0.86 ± 0.24	0.86 ± 0.19		0.9 ± 0.18	0.433, 0.923, 3	0.12, 2.4, − 1.09, 72	0.13, 1.792, − 0.876, 64		0.16, 2.43, − 0.92, 69

*MCA* middle cerebral artery, *ACA* anterior cerebral artery, *PCA* posterior cerebral artery, *VB* vertebral artery, *PSV* Peak Systolic velocity, *EDV* End Diastolic Velocity, *PI* pulsatility Index, *mean* mean flow velocity, P value is significant < 0.05, *the significant between each group with other.

Table 4 showed the comparison between each group of Headache with each other. The TTH group had significantly lower PSV of the MCA and PCA than the group of migraine with aura. In addition, TTH had a significantly lower PSV and MFV of the MCA and a lower EDV in the VA than the group migraine without aura. There was no significant differences between migraineurs with, versus without, aura in any of the parameters.

**Table 4:** Differences in the Resting duplex parameters of anterior and posterior cerebral circulations between different types of headaches (tension-type headache, migraine with aura and migraine without aura).

Artery	Parameters	Tension headache (24 cases)	Migraine with aura (14 cases)	Migraine without aura (23 cases)	Tension type vs migraine with aura	Tension type vs migraine without aura	Migraine with aura vs without aura
		Mean ± SD			P value, F, T, DF		
MCA	PSV	68.9 ± 1.73	85.7 ± 2.8	81.9 ± 1.96	0.043*, 4.78, 0.3, 38	0.02*, 0.26, 2.35, 43	0.64, 4.78, 2.34, 38
	EDV	31.8 ± 10.5	38.9 ± 1.45	36.6 ± 9.4	0.104,4.39,1.8, 38	0.11, 0.01, 1.60, 43	0.58, 4.39, 1.80, 38
	Mean	44.2 ± 12.4	55.0 ± 18.4	50.2 ± 16.1	0.064, 4.5, 2.1, 38	0.04*, 0.17, 2.03, 43	0.60, 4.54, 2.102, 38
	PI	0.78 ± 0.12	0.89 ± 0.10	0.84 ± 0.25	0.58, 1.29, − 0.45, 38	0.74, 1.76, − 0.31, 43	0.45, 1.29, − 0.45, 38
ACA	PSV	60.4 ± 13.8	58.3 ± 1.57	64.01 ± 1.26	0.67, 0.02, − 0.44, 38	0.37, 0.31, 0.90, 43	0.25, 0.02, − 0.44, 38
	EDV	27.0 ± 8.7	23.7 ± 4.7	27.42 ± 6.74	0.10,5.83, − 0.48, 38	0.92, 2.44, 0.08, 43	0.06, 5.83, − 1.48
	Mean	38.5 ± 9.7	35.2 ± 7.8	39.6 ± 7.7	0.28, 0.566, − 0.05, 38	0.61, 1.33, 0.50, 43	0.10, 0.56, − 1.05, 38
	PI	0.90 ± 0.22	0.83 ± 0.18	0.91 ± 0.21	0.25, 1.12, − 0.12, 38	0.91, 0.25, 0.11, 43	0.21, 1.12, − 1.12, 38
PCA	PSV	50.4 ± 1.29	58.9 ± 1.21	55.98 ± 8.54	0.04*, 0.003, 2.09, 38	0.09, 1.31, 1.68, 43	0.41, 0.003, 2.09, 38
	EDV	23.3 ± 7.2	27.1 ± 7.7	26.29 ± 6.5	0.11,0.15, 1.6, 38	0.14, 0.001, 1.46, 43	0.72, 0.15, 1.62, 38
	Mean	32.3 ± 8.9	38 ± 9.19	36.1 ± 6.7	0.05, 0.247, 1.99, 38	0.10, 0.81, 1.62, 43	0.48, 0.25, 1.99, 38
	PI	0.81 ± 0.22	0.811 ± 0.12	0.81 ± 0.16	0.99, 3.6, 0.003, 38	0.95, 1.44, − 0.06, 43	0.93, 3.64, 0.003, 38
VA	PSV	46.1 ± 12.8	48.3 ± 9.96	51.8 ± 1.51	0.55, 0.96, 0.57, 38	0.18, 0.94, 1.36, 43	0.40, 0.96, 0.57, 38
	EDV	19.4 ± 4.9	20.8 ± 3.6	23.2 ± 6.9	0.31, 1.28, 0.96, 38	0.04*, 2.84, 2.15, 43	0.17, 1.28, 0.96, 38
	Mean	28.3 ± 6.7	30.1 ± 4.8	32.7 ± 9.1	0.15, 1.39, 1.35, 38	0.07, 1.70, 1.87, 43	0.44, 1.39, 1.35, 38
	PI	0.86 ± 0.24	0.86 ± 0.19	0.9 ± 0.18	0.90, 0.39, 0.11, 38	0.48, 0.93, 0.69, 43	0.57, 0.39, 0.11, 38

*MCA* middle cerebral artery, *ACA* anterior cerebral artery, *PCA* posterior cerebral artery, *VB* vertebral artery, *PSV* Peak Systolic velocity, *EDV* End Diastolic Velocity, *PI* pulsatility Index, *mean* mean flow velocity, P value is significant < 0.05, *the significant between each group with other.

## Discussion

Few studies have examined cerebrovascular function in tension headache type versus control or versus migraine headache. The present results showed that changes in cerebrovascular function are present both in patients with TTH and in patients with migraine without aura. The former had a low flow velocity of the MCA whereas the latter had a high flow velocity in the ACA.

Compared with the control group there was a significantly lower PSV and MFV of the MCA of the chronic TTH group. This is consistent with the theory of cerebrovascular alteration in chronic TTH with predominant involvement of the MCA. Drummond^26^ reported that exercise-induced changes in the amplitude of temporal artery pulsation were smaller in patients with TTH than in a healthy control group. The authors suggested that this was due to extracranial vasoconstriction.

In contrast there have been reports of a higher time-averaged mean velocity (TAMV) in episodic TTH patients than in controls^31^. Wallasch also reported increased cerebral blood flow velocities (CBF) velocities in the MCA, ACA, and PCA as well as decreased PI in patients with episodic TTH, although there was no difference between controls and patients with chronic TTH^32^. Our results also differ from those of Ozkalayci et al.^2^ They reported a significant increase in CBF in the basilar artery relative to controls (*p* > 0.001).

The data showed a higher MFVin the ACA of patients with migraine without aura than controls, a finding that may support the arteriolar vasodilatation theory in migraine without aura. Abernathy et al.^33^, Fiermonte et al.^18^, and Kastrup et al.^8^ found migraineurs to have higher MFV in the anterior circulation, while others found migraineurs to have higher MFV in the posterior circulation compared to controls^11,34^. However, many previous studies failed to find differences in MFV in either the anterior or posterior circulation between migraineurs and controls^19,24,31,35–46^.

It has been hypothesized that repeated episodes of migraine may alter cerebrovascular function through repeated exposure to neurogenic inflammation, plasma protein extravasation and the release of vasoactive neuropeptides during migraine episodes. If so, abnormalities of cerebrovascular function may be expected to be more evident in migraine patients without aura than controls^35,41^. Another potential explanation is linked to the observation of Lagrèze et al.^47^ who found major abnormalities in rCBF in the grey matter of migraineurs that was normalized by treatment with a calcium entry blocker that prevents migraine attacks. It is therefore possible that the alteration of rCBF or flow velocity could reflect instability of vascular tone especially in migraine without aura. Nevertheless, it remains to be explained whether the modifications of vascular tone are chronic or are an expression of transient abnormalities not associated with headache attacks.

The chronic TH group had lower PSV in the MCA and PCA than the group of migraine with aura. They also had a significantly lower PSV and MFV of the MCA as well as a lower EDV in the VA than in the group of migraine without aura. These results suggest cerebrovascular function in patients with chronic TH differs from that in migraine with or without an aura. Arjona et al.^31^ found a higher TAMV in the MCA in patients who had migraine without aura than in episodic TH. Cerebral autoregulation describes the process whereby cerebral blood flow is maintained constant over a wide range of cerebral perfusion pressure, often from 50 to 150 mmHg in healthy adults. Interestingly, Reinhard et al.^40^ reported that migraineurs with aura have poorer autoregulation in the cerebellar circulation than controls.

In the present study we found no significant difference between migraineurs with or without aura. Asghar et al.^48^ reported that patients who had migraine with aura had a lower blood flow at an earlier stage of the disease than those who had migraine without aura. They suggested that hypoperfusion, or decreased cerebral blood flow, during the aura phase could account for this finding^48^. However, Zaletel et al.^49^ postulated that increased neuronal excitability and neurovascular coupling in migraine could be linked with reduced arteriolar resistance and increased regional blood flow (rCBF)^50^ which can result in the increased flow velocity in the insonated large arteries^51^.

The data of De Benedittis et al.^52^ are consistent with the results of the current study since they found that no significant rCBF difference between migraine with and without aura using single photon emission computed tomography (SPECT) and TCD as measured during headache free-intervals and spontaneous/histamine-induced attacks.

The PI is a non-dimensional parameter that is calculated from the doppler wave form and approximates the value of peripheral resistance to flow (although it can be affected by other hemodynamic factors). Thie et al.^9^ and Fiermonte et al.^18^ reported a lower PI in the cerebral arteries of patients with migraine than in controls. In contrast our data showed no significant changes in PI in any of the three patient groups compared with the control group or between tension headache versus migraine with or without aura.

Silvestrini et al.^53^ suggested that a lower PI was linked to arteriolar vasodilatation. Many studies used TCD ultrasound to compare PI in the anterior circulation of migraineurs and controls^19,31,40,45,54^. Most of them found no significant difference in PI between migraineurs and controls^40,45,54^. Only two studies Chernyshev et al.^34^ and Totaro et al.^10^ found significantly higher PI in the anterior circulation of migraineurs than controls, whereas one study found that migraine patients had lower PI than controls^18^. Few studies compared PI in the posterior circulation of migraineurs to controls; no significant difference in PI between migraines and controls was reported^2,10,34,40^.

Although previous work has shown that cerebral blood flow is altered in migraineurs, it is unknown if these processes are differentially involved in chronic versus episodic forms of the disease. As far as we know there has been no previous study directly comparing episodic versus chronic types of headache. However the present study found no significant differences between episodic and chronic headache in TTH. However, in migraine the PI of the MCA and PCA were significantly higher in patients with chronic versus episodic symptoms. it is possible that a higher frequency of migraine attacks increases PI, which possibly due to arteriolar vasodilatation rather than reduced lumen diameter in the basal arteries.

The present results found abnormal cerebrovascular function in patients with TTH and in migraine without aura. There was a low MFV of the MCA for patients with TTH and a higher MFV of the ACA for patients who had migraine without aura. The increased MFV in migraine without aura may be attributable to arteriolar vasodilatation rather than reduced lumen diameter in the MCA since there was no change in PI.

## Methods

### Participants

The current study was a case–control study, including 61 patients with different types of primary headache: 37 migraineurs, and 24 had TTH headache (≥ 15 attacks /month). The different types of headache were diagnosed according to diagnostic criteria of the international society of headache 2018^55^. They were recruited from the outpatient clinic during the period from 1st January 2019 to 31th December 2019. The migraineurs were classified into 16 had migraine with aura, 21 had migraine without aura, as they are two definite types of primary migraine. Inclusion criteria; both sex were included with age above 20 years old, with duration of headache at least 6 months.

Exclusion criteria: Age below 20 years; patients with acute infarction or hemorrhage, or inability to respond to questionnaires; severe medical illness; vascular disease; diabetes mellitus; addiction; ongoing treatment with calcium blockers (flunarizine) or beta blockers; alcohol abuse. No one received previously Botox injection or calcitonin gene-related peptide (CGRP) inhibitors. Any patient received triptans or ergot drugs as an urgent treatment to abort the attack during study was excluded.

They were compared with a control group of 50 healthy age- and sex-matched volunteers with no history of headache with the same exclusion criteria as in the patient groups.

Demographic and clinical data were obtained for each patient. A full neurological history and neurological examination was obtained in each patient. TCD examination was performed in a quiet room according to previously recommended practice standards. Each participant was examined using TCD ultrasonography of the middle, anterior and posterior cerebral and vertebral arteries (MCA, ACA, PCA, and VA) at rest after temporary discontinuation of any antidepressant or anti-migraineur medication for at least 3 days before the examination according to the exclusion criteria. A CT brain was performed for each patient to exclude secondary headache. All patients were headache-free for at least 3 days at the time of the examination.

The study was approved by the Local Ethics Committee of the Faculty of Medicine, South Valley University and conducted in accordance with the provisions of the Declaration of Helsinki. Written Informed consent was obtained from all participants before enrolment.

### Study procedures

#### Transcranial Doppler ultrasonography

TCD examination was performed in a quiet room according to previously recommended practice standards. All patients were headache free at the time of the examination. TCD employed a SAMSUN HS60 DEVICE made in South Korea with faced array probe 2–4 Hz. Peak systolic velocity, end diastolic velocity, mean flow velocity (MFV) and pulsatile index (PI) for MCA were recorded at depth 40–65 mm, ACA at 60–75 mm, PCA at 55–75 mm, VA at 40–75 mm. The terminals of the MCA, and ACA were defined as anterior circulation, while the VA, and PCA were classified as posterior circulation. Peak systolic velocity (PSV in cm/s): this is the first peak on a TCD waveform from each cardiac cycle. End-diastolic velocity (EDV in cm/s): The end-diastolic flow velocity (EDV) lies between 20 and 50% of the PSV values.

Mean flow velocity (MFV in cm/s): The mean flow velocity is calculated as EDV plus one-third of the difference between PSV and EDV or by (PSV + (EDV*2))/3^1^.

Pulsatility index (PI): Flow resistance is usually assessed by PI, calculated by subtracting EDV from PSV and dividing the value by MFV^1^.

### Analysis

All data were analyzed with the aid of the SPSS ver.16. Descriptive statistics, cross-tabs and frequency tables were used to describe some of the basic variables. A Shapiro–Wilk test confirmed the data was distributed normally so that an independent sample t-test was used to compare continuous variables between groups expressed as mean ± SD. Categorical variables were compared using Fisher’s exact 2-tailed test or by the Chi Square test. One-Way ANOVA was performed between the four groups to determine if there were statistically significant differences in Doppler parameters between the four groups. Bonferroni correction was utilized for two-to-two comparisons when a significant difference was found with ANOVA test. We also re-classified each type of headache (tension-type and migraineurs) into two subgroups: episodic, with less than 15 attacks per month and chronic, with ≥ 15 attacks of headache per month according to the definitions of the International Society of Headache and tested whether this was associated with any difference in flow measures. The accepted significance threshold was p < 0.05.

## Data Availability

Data can be made available to qualified investigators upon reasonable request to the corresponding author.
